# Perfluorocarbon Enhanced Glasgow Oxygen Level Dependent (GOLD) Magnetic Resonance Metabolic Imaging Identifies the Penumbra Following Acute Ischemic Stroke

**DOI:** 10.7150/thno.21685

**Published:** 2018-02-12

**Authors:** Graeme A Deuchar, David Brennan, William M Holmes, Martin Shaw, I Mhairi Macrae, Celestine Santosh

**Affiliations:** 1 Glasgow Experimental MRI Centre, Institute of Neuroscience and Psychology, College of Medical, Veterinary and Life Sciences, University of Glasgow; 2 Department of Neuroradiology, Institute of Neurological Sciences, Southern General Hospital, Glasgow, UK; 3 Aurum Biosciences Ltd, 20-23 Woodside Place, Glasgow, G3 7QL; 4 Clinical Physics, University of Glasgow, Western Infirmary, Glasgow, United Kingdom

**Keywords:** Acute Ischaemic Stroke, perfluorocarbon, penumbra, GOLD metabolic imaging, MRI

## Abstract

The ability to identify metabolically active and potentially salvageable ischaemic penumbra is crucial for improving treatment decisions in acute stroke patients. Our solution involves two complementary novel MRI techniques (Glasgow Oxygen Level Dependant (GOLD) Metabolic Imaging), which when combined with a perfluorocarbon (PFC) based oxygen carrier and hyperoxia can identify penumbra due to dynamic changes related to continued metabolism within this tissue compartment. Our aims were (i) to investigate whether PFC offers similar enhancement of the second technique (Lactate Change) as previously demonstrated for the T_2_*OC technique (ii) to demonstrate both GOLD metabolic imaging techniques working concurrently to identify penumbra, following administration of Oxycyte^®^ (O-PFC) with hyperoxia.

**Methods:** An established rat stroke model was utilised. Part-1: Following either saline or PFC, magnetic resonance spectroscopy was applied to investigate the effect of hyperoxia on lactate change in presumed penumbra. Part-2; rats received O-PFC prior to T_2_*OC (technique 1) and MR spectroscopic imaging, which was used to identify regions of tissue lactate change (technique 2) in response to hyperoxia. In order to validate the techniques, imaging was followed by [^14^C]2-deoxyglucose autoradiography to correlate tissue metabolic status to areas identified as penumbra.

**Results:** Part-1: PFC+hyperoxia resulted in an enhanced reduction of lactate in the penumbra when compared to saline+hyperoxia. Part-2: Regions of brain tissue identified as potential penumbra by both GOLD metabolic imaging techniques utilising O-PFC, demonstrated maintained glucose metabolism as compared to adjacent core tissue.

**Conclusion:** For the first time *in vivo,* enhancement of both GOLD metabolic imaging techniques has been demonstrated following intravenous O-PFC+hyperoxia to identify ischaemic penumbra. We have also presented preliminary evidence of the potential therapeutic benefit offered by O-PFC. These unique theranostic applications would enable treatment based on metabolic status of the brain tissue, independent of time from stroke onset, leading to increased uptake and safer use of currently available treatment options.

## Introduction

Recent data from the global burden of disease 2013 study reveal that every year ~6.5 million people die as a result of stroke worldwide 1 making it the second leading cause of death 2. A further 5 million people worldwide who survive stroke are left permanently disabled with significant long term clinical and social impact on patients lives (http://www.worldstrokecampaign.org). The global burden of stroke continues to escalate with an ageing population being associated with a 62% increase in global death rate between 1990 - 2013 3. This highlights the need for improvements in the management of stroke patients in order to tackle this growing unmet medical need.

The restoration of blood flow through thrombolytic therapy by administration of recombinant tissue plasminogen activator (rtPA) to break down the clot during the first 4.5 h from stroke onset has resulted in benefit for acute ischaemic stroke (AIS) patients 4. The recent emergence of successful mechanical thrombectomy, which utilises clot retrieval devices to remove the obstruction from proximal cerebral arteries thereby restoring blood flow, represents the first major advance in treatment options for acute ischaemic stroke in last 20 years 5,6. However at present this is only suitable in selected patients and requires rapid access to specialist neuorinterventional centres, meaning that clinical use is currently limited. In both instances the benefit gained from successful reperfusion is due to the presence of hypoperfused, metabolically active ischaemic penumbra tissue, the salvage of which is associated with a better outcome 7.

Overall current treatment rates for AIS remain poor with ~75% of patients being excluded from thrombolytic treatment where stroke onset time is unknown, has exceeded 4.5hours or where there is risk-benefit uncertainty. However, many patients excluded on the basis of information on stroke onset time are likely to have potentially salvageable penumbral tissue 8. Of further concern, treatment decisions based largely on time could result in some patients being treated by thrombolysis within 4.5 h who have little or no penumbra, thereby placing patients at increased risk of intracranial haemorrhage with little or no potential for clinical benefit.

Accurate identification of the salvageable ischaemic penumbra is crucial for improving treatment decisions in AIS patients. However, the current lack of an easily applied and accurate imaging technique enabling reliable identification of salvageable penumbral tissue acutely following stroke has hampered progress in attempts to improve the management of patients.

Currently, non-contrast computed tomography is used to quickly evaluate suspected stroke patients to discriminate between ischaemic and haemorrhagic stroke and to exclude stroke mimics 9. However it is insensitive to early changes associated with brain ischaemia and offers no insight into the existence or absence of potentially salvageable penumbral tissue.

Current advanced brain imaging techniques including MRI-based perfusion/diffusion (DWI/PI) mismatch and CT perfusion (CTP) lack accuracy and do not provide direct assessment of tissue metabolic status. Both techniques have already been applied to select patients for acute stroke clinical trials based on “penumbra” imaging. However neither technique, which rely on selecting threshold values to define the DWI lesion and the perfusion deficit, have as yet been validated and accepted for clinical use as routine for penumbra imaging. This lack of validated thresholds leads to difficulties in accurately differentiating penumbra from irreversibly damaged core and benign oligaemic tissue, destined to survive 10,11,12. There is no practical method that gives information on ischaemic brain tissue's ability to metabolically use oxygen, as aerobic metabolism is the critical indicator of whether the penumbra will survive. The availability of new improved diagnostic and therapeutic options for the management of acute ischaemic stroke represents an urgent clinical need.

Our group have been developing GOLD (Glasgow Oxygen Level Dependent) diagnostic imaging, which comprises two independent but complementary MRI based techniques combined with therapeutic potential to extend the lifespan of penumbral tissue. The MRI sequences when combined with an oxygen challenge (OC; 100% inhaled oxygen) identify the ischaemic penumbra by identifying aerobic metabolism within the hypoperfused ischaemic tissue compartment 13,14. Technique 1 utilises a Blood Oxygen Level Dependent (BOLD) T_2_* signal based on the different magnetic properties of deoxy- and oxyhaemoglobin in blood (paramagnetic and diamagnetic, respectively) and this technique has been shown to identify a region within the perfusion deficit in rodent stroke models that displays several features of penumbra: histological evidence of normal neuronal morphology, ongoing glucose metabolism, and tissue recovery on prompt reperfusion 13,15,16. This T2*OC technique has already shown potential for clinical translation in stroke patients 17. However, signal-to-noise was poor due to the limitation in the amount of oxygen that could be delivered to the tissues and the presence of 100%O_2_ within the paranasal sinuses which caused artefacts of T2*-weighted images, due to the paramagnetic effect of oxygen. The technique has also been successfully reproduced pre-clinically by another group 18.

Technique 2 uses the novel concept of lactate change imaging, which can compartmentalise brain tissue based on the ability to identify tissues displaying anaerobic metabolism but having the potential for aerobic metabolism with increased oxygen delivery 14 therefore indicating the tissue as potentially salvageable. Proof of principle for lactate change imaging was also demonstrated with an OC of 100% oxygen alone but again there were issues regarding sensitivity and time required to produce lactate change 14, limitations associated with the amount of oxygen that could be delivered to the tissues using hyperoxia alone.

To increase the amount of oxygen delivered to the tissues we have continued to develop GOLD imaging using an approach where an intravenous oxygen carrier perfluorocrabon (PFC) is administered prior to OC. In addition, unlike other advanced brain imaging techniques, additional imaging time carries no penalty (“time lost is brain lost”) 19 since PFC+oxygen has the potential to maintain brain viability and reduces ischaemic damage. GOLD diagnostic imaging would replace a reliance on the time clock and replacing it with a metabolic tissue clock based on identifying patients who still have a significant volume of penumbra, so that many more patients could benefit from current treatments. It would also enable identification of patients who do not have existing penumbra within current treatment windows for acute stroke, where the benefit-risk balance doesn't justify the use of thrombolysis thus avoiding the unnecessary risk of fatal haemorrhage.

Perfluorocarbons (PFCs) are fluorinated hydrocarbons with respiratory gas carrying capacity superior to that of haemoglobin 20. They are hydrophobic in nature and therefore have to be emulsified for intravenous use. PFC particle size within emulsions is very small (~0.2 μm compared to ~7 μm for erythrocytes), enabling better penetration through the microcirculation (capillary size ~7 μm) improving oxygen supply to poorly perfused tissue. These characteristics coupled with the release of oxygen by simple diffusion down a partial pressure gradient make PFCs ideal agents by delivering more oxygen to the ischaemic tissues and so overcoming the current limitations of translating both GOLD imaging techniques for diagnostic evaluation in the acute stroke setting. Having initially designed and produced our own bespoke PFC emulsion (b-PFC), which was used in study 1 we subsequently established a strategic collaboration to develop Oxycyte® (O-PFC) intravenous emulsion, which is a clinical stage PFC emulsion. This would accelerate the translation of our acute stroke programme as this product has already undergone significant pre-clinical safety/toxicology and early human trial work in traumatic brain injury.

We have previously demonstrated enhanced detection of penumbra with T2*OC using our b-PFC combined with a lower level (40%) of inhaled oxygen 21. Therefore, the addition of PFC to OC has demonstrated potential to overcome the limitations encountered in early clinical studies.

The primary aims of the current studies were to (i) determine whether our lactate change technique could also be enhanced by accelerating the change with the addition of intravenous PFC (ii) investigate the potential of combining the metabolic GOLD imaging techniques (T2*OC and Lactate Change) within a single scanning protocol, with confirmation of ongoing glucose metabolism in regions identified as penumbra using [^14^C]2-deoxyglucose autoradiography. A secondary aim was to investigate the therapeutic potential of the enhanced oxygen delivery to the compromised penumbra by addition of intravenous PFC in combination with hyperoxia.

## Materials & Methods

### Model of Middle Cerebral Artery Occlusion (MCAO)

Experiments were performed under license from the UK Home Office, were subject to the Animals (Scientific Procedures) Act, 1986 and were approved by the University Ethical Review Panel. Experiments have been carried out and reported in accordance with the ARRIVE (Animal Research: Reporting In Vivo Experiments) guidelines. Male Sprague Dawley rats (327 ± 26 g, n=24), Harlan, Bicester, UK) had free access to food and water and were maintained on a 12 h light-dark cycle. Isoflurane (5%) in N_2_O:O_2_ (70%:30%) was used for induction of anaesthesia and, following tracheostomy, was lowered during further surgery (2.5-3%) and maintenance of animal throughout the protocol (2-2.5%). N_2_O:O_2_ was replaced with medical air supplemented with ~5% O_2_ (26% O_2_: normoxia) prior to middle cerebral artery occlusion (MCAO). Animals were artificially ventilated and body temperature maintained at 37°C. Femoral arteries were cannulated (Portex: external diameter 0.96 mm; internal diameter 0.58 mm) for mean arterial blood pressure (MABP) measurement and blood gas analysis in order to maintain physiological stability. A femoral vein was cannulated for administration of the PFC emulsion or saline. MABP and heart rate were continuously recorded (AcqKnowledge, Biopac Systems, CA, USA). Permanent MCAO was induced with an intraluminal filament. A 3.0 uncoated nylon filament with a heat-induced bulb at the tip (~320-340 µm in diameter) was advanced along the left internal carotid artery for ~20-21 mm from the bifurcation of the external carotid artery until resistance was felt. Following MCAO rats were transferred, under the same anaesthetic, directly into the magnet bore, fully instrumented for monitoring and control of physiological variables (blood pressure, temperature and blood gases). Successful MCAO was confirmed from the initial diffusion-weighted imaging (DWI) and perfusion imaging (PI) MRI scans.

Although permanent MCAO (pMCAO) may not be representative of many ischaemic stroke patients it represents the best model for these preclinical proof of concept studies developing and validating the diagnostic techniques used for identifying penumbra. The pMCAO model displays less heterogeneity in the initial lesion size when compared to the transient filament or embolic MCAO models also utilized by our group. In our experience this model shows evidence from previously published work of a significant amount of penumbral tissue, which gradually becomes incorporated into the ischaemic core over the first 4-6 h following stroke 13,15,16.

### Magnetic Resonance Imaging Scanning

MRI was performed on a Bruker Biospec 7T/30 cm system (Bruker Biospin, Ettlingen, Germany) equipped with an inserted gradient coil (121 mm ID, 400 mT/m) and a 72 mm birdcage resonator. Following stroke surgery, the animals were placed in a rat cradle with head secured in position using ear and tooth bars to restrict movement. Brain imaging was acquired using a surface coil placed above the head. For **Study 1** we utilised localised H^1^ Magnetic Resonance spectroscopy (MRS) in combination with DWI and PI using a 2 cm linear surface receiver coil. For **Study 2** (spectroscopic imaging) a 4-channel phased array coil was used which enabled novel spectroscopic imaging of brain lactate levels consecutively with DWI, PI and T_2_*.

#### Diffusion weighted imaging (Study 1 and 2)

DWI was employed to identify acute ischaemic damage: For quantitative determination of the apparent diffusion coefficient (ADC) a 4-shot spin echo planar imaging (EPI) diffusion weighted scan (echo time: 22.5 ms, repetition time: 4000.3 ms, 4 averages, matrix: 96 x 96, FOV: 25 x 25 mm, 3 directions: x, y, z, B values: 0, 1000 s/mm^2^, 8 contiguous coronal slices of 2.0 mm thickness, 4 shot EPI) was used for quantitative determination of the ADC.

#### Perfusion Imaging (Study 1 and 2)

PI was employed to identify the blood flow deficit: Non-invasive, relative cerebral blood flow (CBF) measurements were carried out within the MCA territory using a form of pseudo-continuous arterial spin labelling (ASL) based on a train of adiabatic inversion pulses 22. The sequence employs a spin-echo EPI imaging module (echo time: 20 ms, repetition time: 7000 ms, matrix 96 x 96, FOV 25 x 25 mm, slice thickness 2.0 mm, 16 averages, 4 shots) preceded by 50 hyperbolic secant inversion pulses in a 3 second train.

#### Localised Magnetic Resonance Spectroscopy (Study 1)

Localised ^1^H spectra were acquired using a point resolved spectroscopy sequence (PRESS) (echo time, 20 ms; repetition time, 3000 ms). Voxels of 4 x 4 x 4 mm^3^ were placed in the ischemic region, defined by DWI and homotopic contralateral caudate nucleus. Spectra were acquired with 16 averages. A smaller voxel 2.5 x 2 x 4 mm^3^ was placed in the cortical DWI/PI mismatch region. To increase the signal noise ratio, 90 averages were acquired. The time taken to acquire a block of three spectra for the three distinct regions of interest (ROIs) was 10 minutes.

#### Spectroscopic Lactate Imaging (Study 2)

Spectroscopic imaging of lactate was performed using a novel pulse sequence specifically designed to efficiently image changes in lactate within the brain 14. For the current study, this sequence was designed to acquire data from a single slice, although it should be adaptable to multi-slice acquisition for future development. A three-pulse CHESS module was used for water suppression. For excitation, a frequency selective 6 ms Gaussian pulse was used with a bandwidth of 452 Hz centered at 1.06 ppm. This frequency range includes lactate, lipid and macromolecular resonances, and as such, acquires a composite image. As the frequency selective pulse excites lipids outside of the brain (e.g. subcutaneous fat), it is necessary to use outer volume suppression around the brain to avoid saturating the signal of interest from within the brain. The selected signal was then imaged using a RARE imaging module (echo time, 14.5 ms; repetition time, 2800 ms, 256 averages; field of view, 1.5_2.0 cm^2^; 42 x 64 matrix; resolution, 357 mm x 390 mm; slice thickness, 2 mm; RARE factor, 32). Scan time was 12 min.

#### T_2_* scanning (Study 2)

The T_2_* sequence was a single shot, gradient echo (EPI) sequence (echo time, 20 milliseconds; repetition time, 10 s; matrix 96_96, field of view, 25_25 mm, 8 contiguous slices, 1.5 mm thick, 2 averages, temporal resolution 20 s, 75 repetitions). Two coronal slices within middle cerebral artery territory were selected to generate T2* signal change maps, which were used to generate the mean signal change for each region of interest (ROI) across the two slices. Penumbra tissue was defined using a threshold based on the empirical rule: tissue displaying a mean increase in T2*, which was greater than two standard deviations above the contralateral cortex mean.

### Perfluorocarbon intravenous emulsion

For Study 1 we used an in-house designed PFC emulsion including perfluorodecalin (C_10_F_18_) (F2 Chemicals Ltd, UK), which is known to have an oxygen solubility of 49 ml/100 ml of aqueous PFC at standard temperature and pressure. This bespoke PFC emulsion (b-PFC) was emulsified for i.v. use in a phosphate buffered solution containing (4% w/v) purified egg yolk lecithin (Lipoid 80 S Egg Lecithin, Ludwigshafen, Germany). The final emulsion for i.v. use was 40% w/v perfluorodecalin and was prepared by Professor Julian Eastoe, an expert in emulsion chemistry at the University of Bristol. The mean droplet diameter within the emulsion, analysed using dynamic light scattering was shown to be similar to that reported in the literature (~0.2µm).

With an objective of progressing into clinical trials investigating the potential of GOLD in acute stroke patients, and recognising the lengthy and costly process of developing a bespoke PFC emulsion as a new pharmaceutical, we entered into collaborative agreements with Tenax Therapeutics Inc (formerly Oxygen Biotherapeutics) to use the PFC emulsion Oxycyte^®^ (O-PFC) in our acute stroke development programme. O-PFC had already undergone significant non-clinical safety assessment and had been used in early human trial work where it progressed into a Phase 2b trial in traumatic brain injury (NCT00908063). Therefore, the use of O-PFC in the ongoing non-clinical activities would offer obvious benefits in expediting the development pathway for our GOLD imaging technology and this was the PFC emulsion used in Study 2 investigating the feasibility of combined lactate change and T2* OC imaging.

### Experimental protocol for Study 1: Localised Spectroscopy to measure Lactate Change in Penumbra

**Figure 1:**
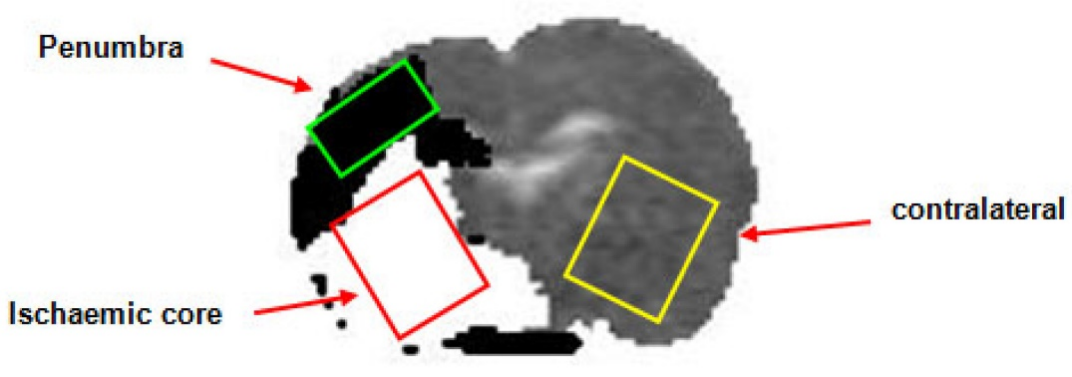
Apparent diffusion coefficient rat brain image illustrating thresholded DWI (white, ischaemic core)/PI mismatch (black, presumed penumbra) with the placement of the voxels for localised MRS (Study 1). DWI: diffusion-weighted imaging; PI: perfusion imaging; MRS: magnetic resonance spectroscopy.

Following transfer into the magnet bore a period of stabilisation (~20 min) was allowed during which arterial blood gases, blood pressure, ECG and respiration were monitored to ensure haemodynamic stability and values within the normal physiological range. Stroke success was confirmed using initial PI and DWI scans with initial perfusion and ADC maps used to position voxels within the regions of interest (ROIs) in the ischaemic core, homotopic contralateral caudate nucleus and DWI/PI mismatch region (approximate penumbra, Figure 1)). Localised ^1^H spectroscopy, was used to investigate changes in the metabolite spectra. Initial baseline spectra were acquired for each voxel with the animal ventilated on air up to a point where there appeared to be an increase in the lactate peak within the penumbra voxel. During this baseline period further DWI scans were acquired, enabling the generation ADC maps at regular intervals. This was done to ensure that the penumbra voxel had not incorporated tissue consistent with ischaemic core.

When an increase in the lactate peak was observed in the penumbra voxel, b-PFC (1.5 mL, n=7) or saline (1.5 mL, n=8) was administered intravenously. The fraction of inspired oxygen (FiO_2_) was subsequently increased to ~100% for 30 minutes during which 3 blocks of spectra were acquired for each ROI voxel. Ventilation was then switched back to normoxia for 30 minutes and a further 3 blocks of spectra acquired for each ROI voxel. ADC maps were acquired at regular intervals during the experiment including at baseline prior to 100% O_2,_ following 30 minutes 100% O_2_ and following 30 minutes with the animal returned to normoxic ventilation. This also allowed for an assessment of change in lesion volume during the experimental protocol.

The spectra were processed individually with TopSpin™ (Bruker Biospin, Rheinstetten, Germany) using automatic phasing and automatic baseline correction. The change in lactate at each time point following both 100% O_2_ and upon returning to normoxic ventilation was determined from the change in the ^1^H spectra integral between 1.2 ppm and 1.9 ppm (measured using Area Under the Curve; AUC).

### Experimental protocol for Study 2: Combined T_2_* and Lactate Change OC GOLD Imaging techniques to identify the penumbra

**Figure 2:**
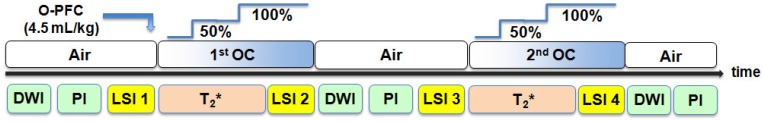
Schematic time line for Study 2 MRI protocol. Baseline scans aquired under normoxic ventilation (AIR) included DWI and PI to confirm stroke and a basal lactate scan (LSI). Following administration of O-PFC, the initial OC (50% O_2_ for 6 min and 100% O_2_ for 6 min) was carried out (88 min ± 13 min post-stroke) during which a T_2_* scan and a further lactate scan was acquired. The animal was subsequently returned to normoxic ventilation with further DWI, PI and lactate scans acquired under these conditions. A second OC was carried out (159 min ± 13 min post-stroke), during which a further T_2_* scan and lacate scan were acquired. DWI: diffusion-weighted imaging; LSI: lactate specroscopic imaging; MRI: magnetic resonance imaging; OC: oxygen challenge; PI: perfusion imaging.

Following permanent MCAO in rats (n=9) animals were transferred into the magnet bore (Bruker 7T Biospec) and there was a period of stabilisation, during which blood pressure, ECG and respiration were monitored to ensure haemodynamic stability and values within the normal physiological range. The MRI scanning protocol is shown in Figure 2. During the initial hour after MCAO, DWI and PI were carried out to confirm the presence of ischaemic injury and cerebral blood flow deficit, respectively. Spectroscopic imaging was used to spatially identify regions where changes in tissue lactate occurred in response to periods of hyperoxia. Following initial DWI/PI scans a water image was acquired using the spectroscopic imaging sequence (bandwidth 452Hz, centered at 4.7ppm) and this was used during analysis for lactate image registration.

The combined T_2_*OC / Lactate Change OC penumbra imaging protocol (Figure 2) was carried out as follows. With the animal on normoxic ventilation, an initial baseline lactate spectroscopic image (single coronal slice) was acquired within middle cerebral artery territory that was predicted from a previous study 21 to match approximately to where the T_2_* signal change maps would be generated. At a mean time of 75 minutes following MCAO, animals were administered 1.5 ml (~4.5 ml/kg, IV) O-PFC as a slow bolus over 6 minutes. Next, the first oxygen challenge T_2_* scan was acquired at a mean time of 86 minutes after stroke onset: following 3 minutes of scanning for baseline (normoxia), O_2_ was increased to 50% for a period of 6 minutes and then increased to 100% for a further 6 minutes. Ventilation was kept on 100% O_2_ and a further lactate spectroscopic image was acquired. The animal was then returned to normoxic ventilation and allowed a period of stabilisation (~20 minutes) to return to normal physiological parameters. This protocol of OC during lactate change spectroscopic imaging and T_2_* was then repeated (mean time for second T_2_* scan was 160 minutes following MCAO. Additional DWI and PI scans were taken throughout the protocol.

#### [^14^C]2-Deoxyglucose (2-DG) in vivo autoradiography

At the end of the MRI scanning session, animals were quickly removed from the magnet, and returned to the operating theatre where a bolus of [^14^C]2-DG was injected intravenously at a steady rate over 30 seconds (125 mCi/kg in 0.6mL heparinized saline, Perkin-Elmer, Waltham, MA, USA). Plasma glucose and [^14^C] were analyzed from 14 timed arterial blood samples over 45 minutes by glucose oxidase assay and liquid scintillation analysis, respectively. At 45 minutes, animals were killed by intravenous injection of sodium pentobarbitone and the brains quickly dissected out, frozen (isopentane, -40°C) and processed for quantitative autoradiography. Coronal cryostat sections (20 µm) were exposed to X-ray film (Kodak Biomax MR film, Eastman Kodak Company, Rochester, NY, USA) for 3 days with a set of [^14^C] standards (Amersham Biosciences, GE Healthcare, Little Chalfont, Buckinghamshire, UK). Autoradiograms were analyzed using a computer digitized image analysis system (MCID v4, Interfocus, Linton, Cambridge, UK). Quantitative optical density measurements were taken from four regions of interest (ROIs defined in detail below with an example shown in Figure 1iii): (1) ischemic core; (2) T_2_* defined penumbra; (3) contralateral region homotopic to the penumbra and (4) contralateral region homotopic to the ischemic core. Optical density values were converted into [^14^C] tissue concentrations using the calibration curve derived from the set of [^14^C] standards. The [^14^C] tissue concentrations along with the plasma glucose and [^14^C] plasma concentrations were used to calculate local cerebral glucose utilization (LCMRglu, mmol per 100 g per minute) in ROIs using the operational equation of Sokoloff 23.

Glucose utilization for each ROI was generated from 3 autoradiographic images covering the rostro-caudal extent of each of the two selected coronal MRI slices (2 mm) used to generate the T_2_* signal change maps, which also approximated the slice used for lactate change maps. This enabled glucose utilization within each ROI to be determined from autoradiograms.

### Image Analysis

ADC maps, CBF maps and T_2_^*^ percentage signal change maps during hyperoxia and lactate spectroscopic images were generated using Image J software (http://rsb.info.nih.gov/ij/).

Lactate Change OC maps were generated for a single coronal slice by subtraction of spectroscopic images acquired during baseline (normoxia) and hyperoxia with a spatial smoothing filter being applied. Aerobic Lactate Change maps represent the change in tissue lactate in response to OC following administration of O-PFC in Study 2. Anaerobic Lactate Change maps represent the change in tissue lactate in response to returning to ventilation with medical air following a period of hyperoxia.

T_2_^*^ percentage signal change was analysed on MRI-based ROIs, which were selected according to specific features on the images; (1) Ischaemic core in caudate nucleus within the thresholded ADC lesion; (2) Its mirror contralateral region manually designated by researcher; (3) Penumbra as defined by thresholded T_2_^*^ percentage signal change (4) contralateral cortex (see Figure 7A iii). In selecting ROIs, the mean T_2_* percentage signal change (with standard deviation) was measured in the dorso-lateral contralateral cortex, avoiding any large veins and venous sinuses. The T_2_* penumbra ROI was automatically derived from a threshold: tissue with a percentage signal change ≥ the contralateral ROI mean + two standard deviations. The ischaemic core ROI was selected as a region within the thresholded ADC lesion for the corresponding slice. A representative contralateral region was manually drawn avoiding areas of high signal change on T_2_* maps due to the presence of large veins, meaning that the region was not always an exact mirror of the ipsilateral ROI.

The ROIs were selected and analysed on two coronal slices within middle cerebral artery territory. T_2_^*^ percentage signal change maps were produced by comparing the peak signal during each level of the hyperoxia protocol (mean taken over ~2 min scanning) with the mean signal during baseline (first 3 min of scan prior to hyperoxia). The difference in signal was divided by the mean baseline signal and multiplied by 100 to give percentage signal change maps. Penumbra tissue was defined using a threshold based on the empirical rule: tissue showing a mean increase in T_2_^*^ which was greater than 2 standard deviations above the contralateral cortex mean.

Approximate penumbral tissue generated from DWI-PI mismatch area (Figure 7A iii) was also used for comparison with the regions detected as penumbra by T2* and Lactate Change OC imaging. This was done by producing quantitative ADC maps in units of square mm per second using the Stejskal-Tanner equation 24. A reduction of 16.5% in ADC when compared to mean contralateral value was used as a threshold to determine ischaemic lesion volume as this has previously been reported to closely match final infarct size in a model of permanent MCAO in Sprague Dawley rats 25. CBF maps were produced to identify the perfusion deficit area which was calculated using a threshold of a 57% reduction of the mean contralateral CBF 26.

### Data presentation and statistics

All data are presented as mean ± SD. Statistical analysis was performed using GraphPad Prism (Version 4.03, CA, USA). Comparison of physiological variables before and during OC, and of OC-induced T_2_*signal change in penumbra, contralateral cortex and ischaemic core ROIs were assessed using a 2-tailed paired Student's t-test. Data from different groups were assessed using a 2-tailed unpaired Student's t-test. For comparison of paired data within groups, such as the change in lactate levels measured within the penumbra voxel from Study 1, a repeated measures one-way ANOVA followed by a Dunnet's multiple comparison test was performed. For comparison of unpaired data such as the magnitude of T_2_* signal change between ROI's, a one-way ANOVA followed by a Bonferroni's multiple comparison test was performed. For all comparisons, p<0.05 was considered as statistically significant.

## Results

### Study 1 Localised Spectroscopy

#### Physiological Variables

Physiological data are shown in supplementary Table S1. Prior to administration of either saline or b-PFC and the subsequent OC, physiological variables were within the normal physiological range. During both saline and b-PFC injection there was a small, significant increase in MABP, which had returned to a level not different from baseline prior to initiation of OC. Oxygen challenge (FiO_2_ ~1.0) induced a transient, significant increase in PaO_2_ of ~3.5-4.0 fold in arterial blood samples from both groups. This is consistent with previous studies using this level of hyperoxia 13,27. There was a small, significant increase in PaCO_2_ observed during OC in the group receiving saline, prior to hyperoxia.

#### Prior administration of b-PFC Improves Sensitivity of Lactate Change OC Imaging

**Figure 3:**
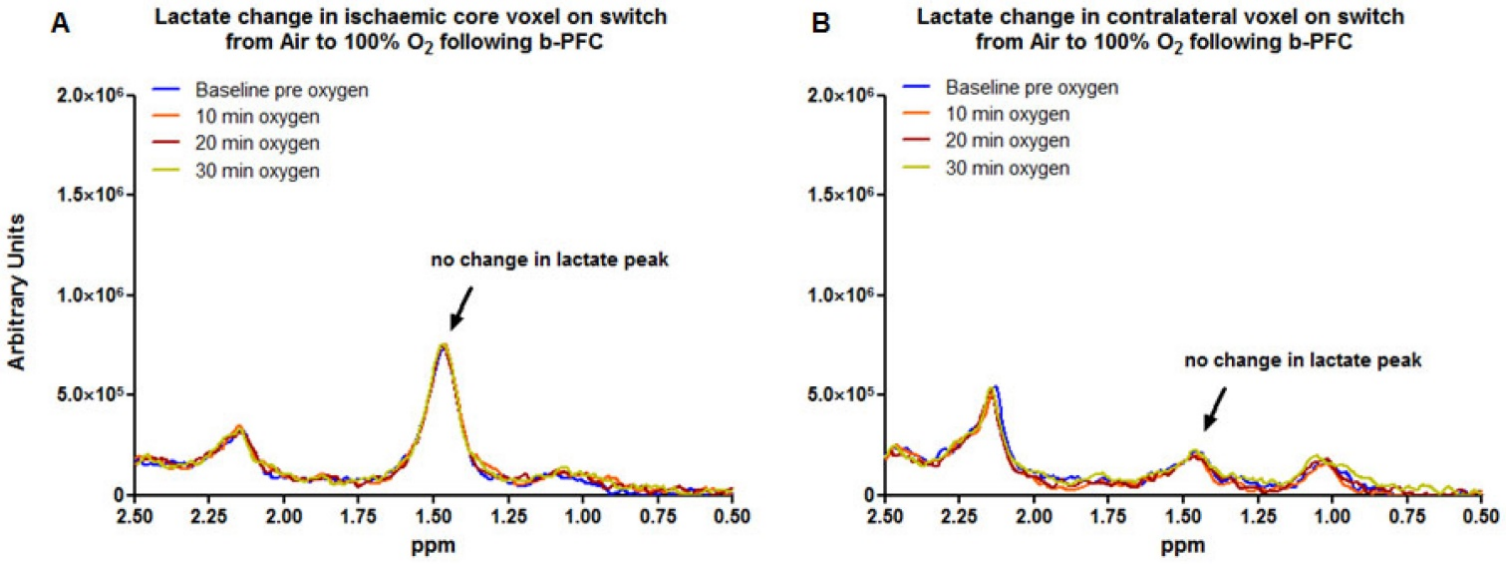
Representative examples of localised 1H spectra from (A) the ischaemic core voxel and (B) the corresponding region of the contralateral hemisphere following MCAO in an animal administered b-PFC (4.5 mL/kg, i.v.). MCAO: middle cerebral artery occlusion.

Data acquired from the ischaemic core (Figure 3a) and contralateral caudate nucleus (Figure 3b) demonstrated no change in the lactate peak on the ^1^H spectra upon changing ventilation from air to 100% oxygen in animals receiving either PFC or saline prior to hyperoxia (representative spectra are shown in Figure 3).

**Figure 4:**
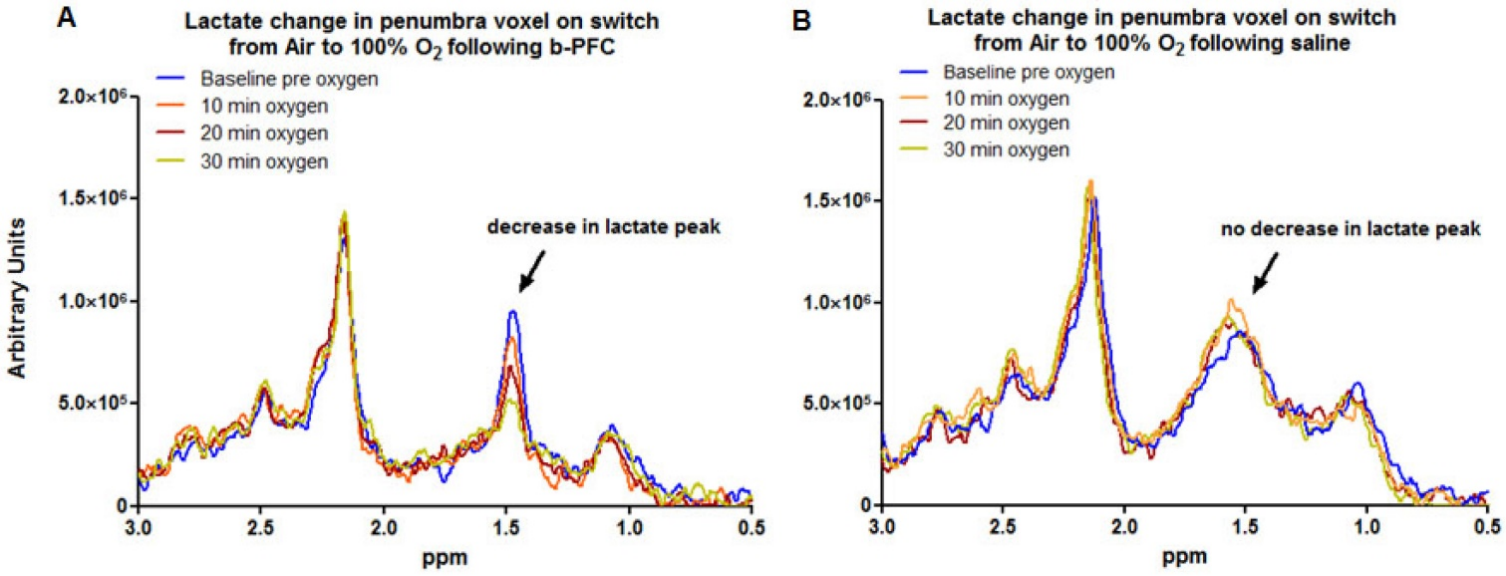
Representative examples of localised 1H spectra from (A) the penumbra voxel in an animal administered b-PFC (4.5 mL/kg, i.v.) and (B) the penumbra voxel in an animal administered saline (4.5 mL/kg, i.v.) following MCAO. MCAO: middle cerebral artery occlusion.

**Figure 5:**
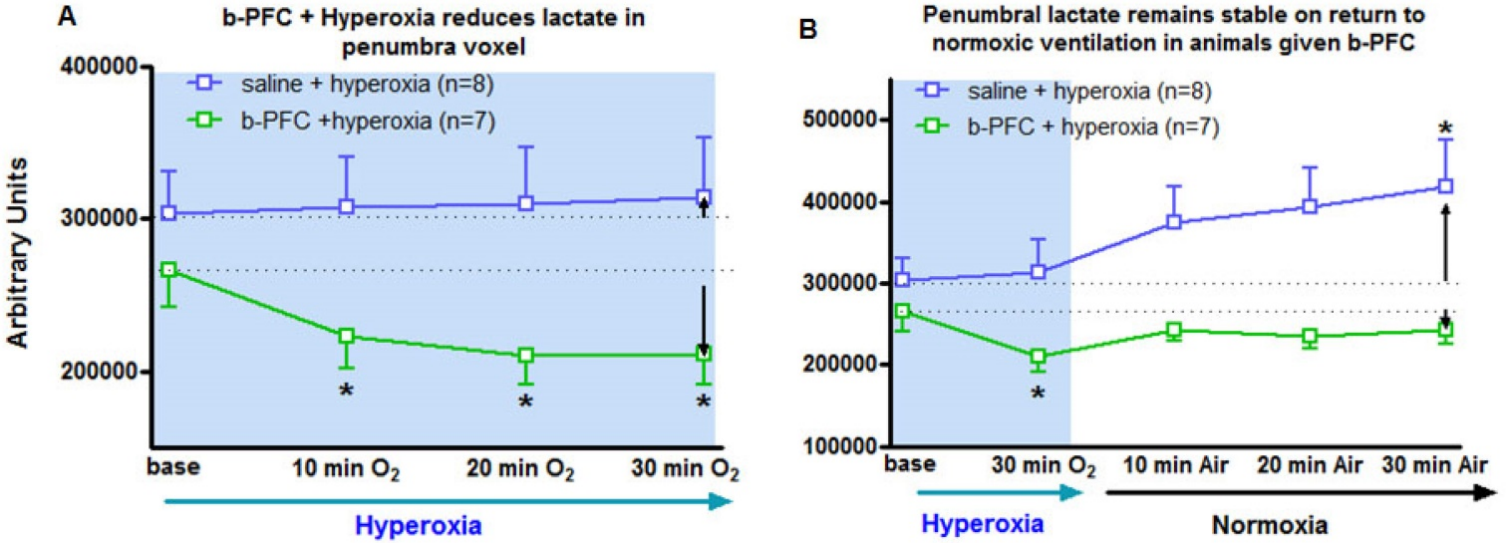
Group mean data (with SD error bars) for (A) 30 min hyperoxia (100% O_2_) on lactate levels following administration of either saline (4.5 mL/kg, i.v.) or b-PFC (4.5 mL/kg, i.v.). (B) Same data extended to include the effect of a return to normoxic ventilation. *p<0.05 versus corresponding group base using one-way repeated measures ANOVA followed by Dunnett's multiple comparison test.

In contrast, in the penumbra voxel a reduction in the lactate peak was consistently observed when ventilation was switched from air to 100% oxygen in animals receiving 4.5 ml/kg b-PFC prior to hyperoxia (Figure 4A, 5A). However, in animals receiving saline, the effect on the lactate peak was more variable with further increases in the lactate peak observed in some cases (Figure 4B, 5A).

Penumbra lactate levels increased by 3.5% during 30 min hyperoxia in the control saline group compared with a maximum decrease of 20.8% in the b-PFC group (Figure 5A).

When ventilation was switched back to air (Figure 5B) the lactate peak significantly increased by a further 32.4% within 30 min in the saline group, while the decrease in lactate in the b-PFC group was maintained below pre-hyperoxia baseline values.

**Figure 6:**
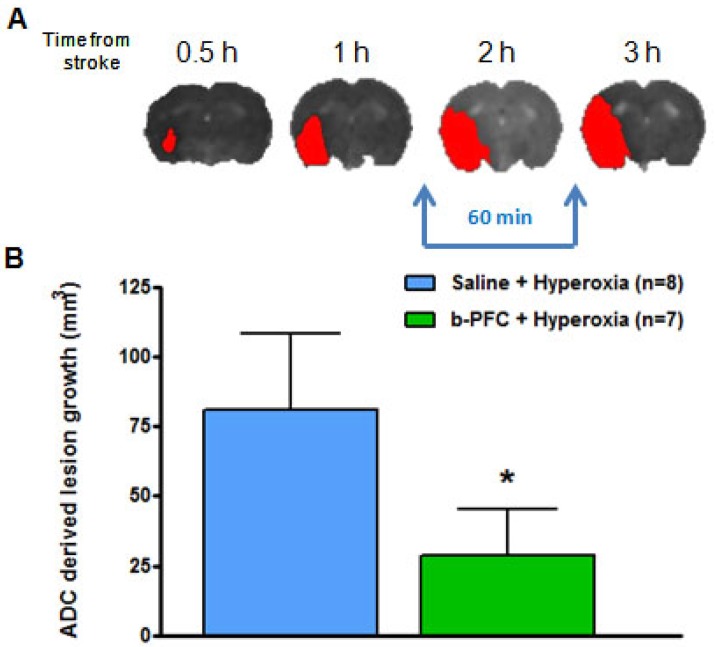
(A) Illustrative example showing ischaemic damage defined from thresholded ADC maps (red) progresses with time. (B) Growth of ischaemic damage assessed over 60 min starting ~ 90 min after stroke. Rats received either 4.5 mL/kg b-PFC or saline followed by 30 min hyperoxia (100% O_2_) then normoxia for 30 min. Data displayed as mean ± SD, *p<0.05. ADC: apparent diffusion coefficient.

The ADC derived lesion volume measured over the same period revealed significantly less lesion expansion in the b-PFC group (28.8 ± 16.7 mm^3^) compared to the saline group (80.8 ± 27.3 mm^3^) (Figure 6).

### Study 2: Combined T_2_* and Lactate change OC GOLD Penumbra Imaging

#### Physiological Variables

Physiological data were within the normal physiological range prior to administration of O-PFC or saline and initiation of OC (supplementary Table S2). During administration of O-PFC there was a transient (~10%) increase in MABP. Following the completion of O-PFC administration MABP decreased below the pre-injection baseline but remained within the normal range. During hyperoxia, at both early and later time points, there was a similar increase in MABP (~15%) during 50% O_2_ OC with no further increase evident on increasing oxygen to 100%. Similar to Study 1 results, an approximate 4 fold increase in PaO_2_ was evident in arterial blood samples following 100% OC. No significant changes were evident in PaCO_2_ during hyperoxia.

#### Demonstration and Validation of Penumbra Detection Utilising OC T2* and Lactate Change GOLD Imaging Concurrently

**Figure 7:**
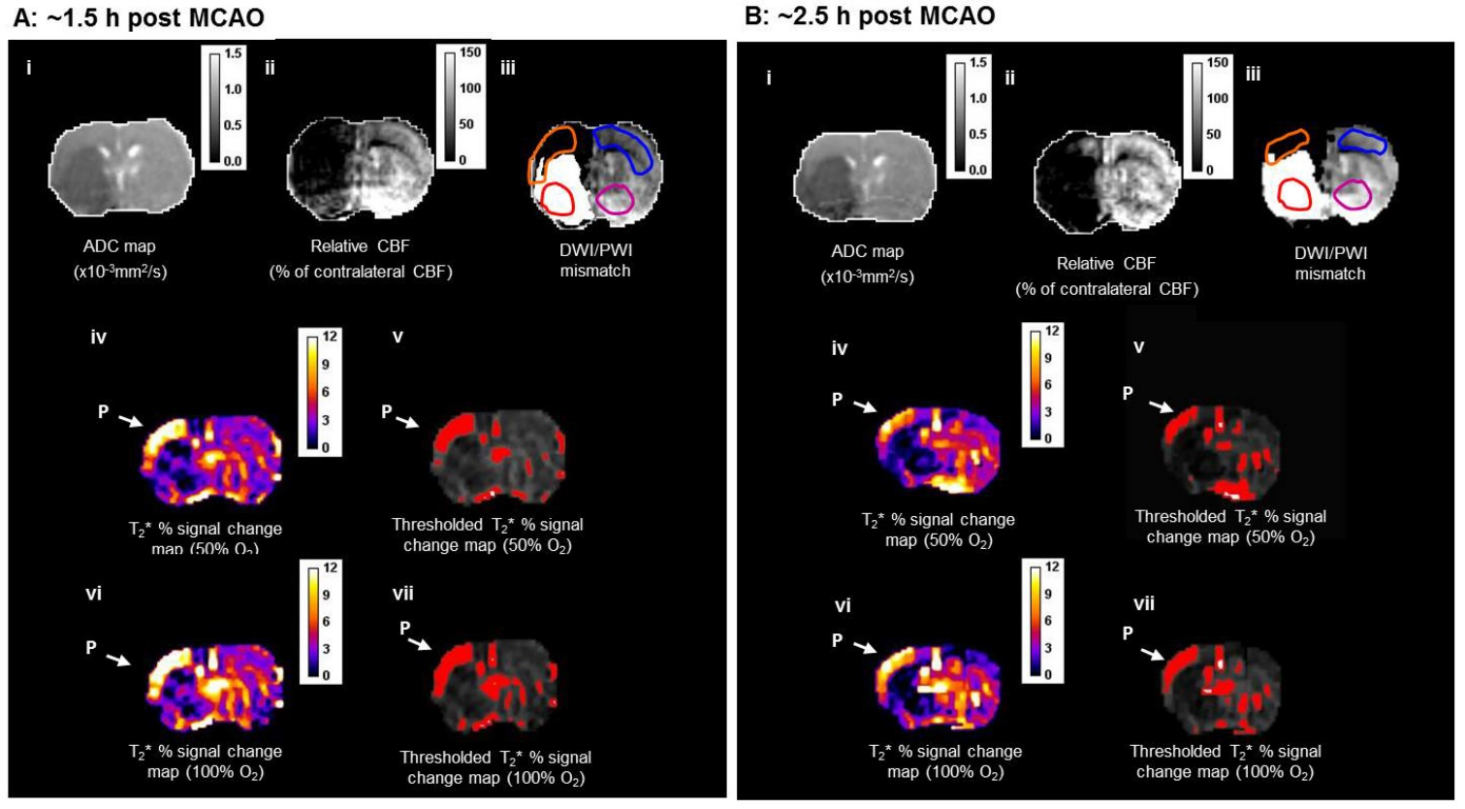
Detection of penumbra from a representative animal using perfusion (ii) diffusion (i) mismatch (iii), and T2*OC (iv, v, vi and vii) at ~1.5 h (A) and ~2.5 h (B) following MCAO with prior administration of O-PFC (4.5 mL/kg). (i) ADC map prior to initial oxygen challenge; (ii) relative CBF map for the same slice and (iii) the corresponding DWI/PI mismatch (black shading) with selected ROIs superimposed (orange T2*OC penumbra; blue, mirror contralateral ROI; red, ischaemic core; and purple, mirror contralateral ROI). (iv) Thresholded T_2_*OC signal change map (%) using 50% O_2_ with (v) the corresponding thresholded image. (vi) and (vii), corresponding images using 100% O_2_. P = penumbra. ADC: apparent diffusion coefficient; CBF: cerebral blood flow; DWI: diffusion-weighted imaging; MCAO: middle cerebral artery occlusion; OC: oxygen challenge; PI: perfusion imaging; ROI: region of interest.

DWI/PI mismatch tissue (Figure 7A iii) was derived from coregistration of thresholded ADC (Figure 7A(i)) and CBF maps (Figure 7A(ii)) and used for comparison with penumbra identified using both T_2_* and lactate change OC techniques.

##### Detection of T2* OC defined penumbra following O-PFC

**Figure 8:**
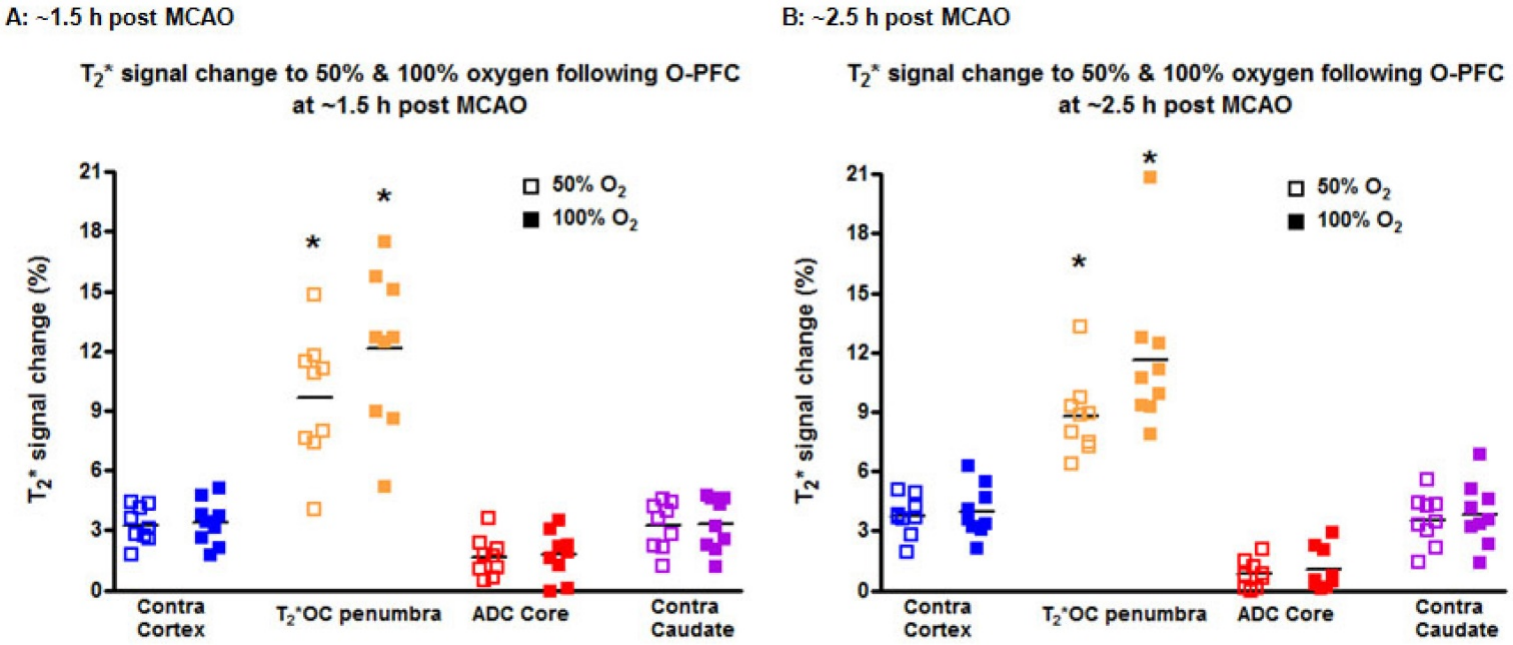
T_2_* signal change to 50% and 100% oxygen challenges for selected ROIs carried out at **(A)** ~1.5 h and **(B)** ~2.5 h following MCAO. O-PFC was administered just prior to the initial oxygen challenge. Bars indicate means. * indicates significantly greater signal change (p<0.05) in T_2_*OC defined penumbra compared to all other ROI using one-way ANOVA followed by Bonferroni's Multiple Comparison Test. Contra cortex, contralateral cortex; T_2_*OC penumbra, T_2_* oxygen challenge-defined penumbra; ADC core, ADC-defined ischaemic core; Contra caudate, contralateral caudate nucleus. ADC: apparent diffusion coefficient; MCAO: middle cerebral artery occlusion.

During the initial OC (~1.5 h post-MCAO) the magnitude of T_2_* signal change during both 50% and 100% OC varied throughout the selected ROIs (Figure 7A, iv and vi). In the ipsilateral hemisphere, the region defined as ischaemic core by thresholded ADC maps showed the smallest percentage change, while the thresholded T_2_*-defined penumbra displayed significantly greater changes during 50% and 100% OC (Figure 7A iv & vi, Figure 8A). When compared to the contralateral cortex, the thresholded T_2_*-defined penumbra displayed significantly greater changes to both levels of hyperoxia (Figure 7A iv & vi, Figure 8A). Following a period when the animals were returned normoxic ventilation, similar results were evident during the second OC (~2.5 h post-MCAO, Figure 7B & 8B) with once again, significantly greater signal change evident in the T_2_*-defined penumbra ROI when compared to all other ROIs.

**Figure 9:**
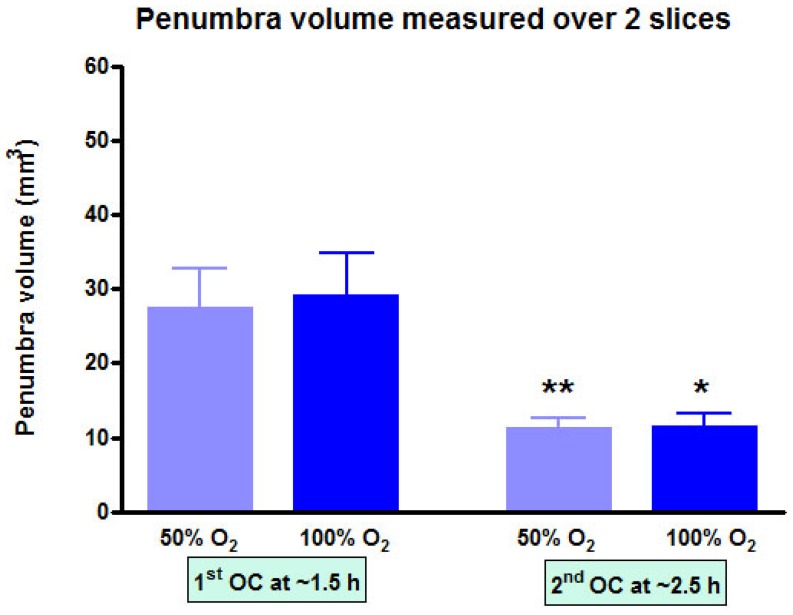
Penumbra volume measured across the two slices (slice thickness, 1.5 mm) used to analyse T_2_*OC responses. *p<0.05 **p<0.01. Indicates a significant reduction in penumbra volume from 1.5 h to 2.5 h post-MCAO. Data presented as mean ± SD, n=9. MCAO: middle cerebral artery occlusion.

At each time point, the volume of penumbra defined by T_2_*OC with O-PFC was similar for 50% and 100% OC (Figure 9). The animals were ventilated on air between the two OCs (normoxia) and it was notable that penumbra volume at ~2.5 h post-MCAO was significantly smaller than that detected at the earlier time point (Figure 9).

##### Simultaneous detection of penumbra from lactate change maps following O-PFC

**Figure 10:**
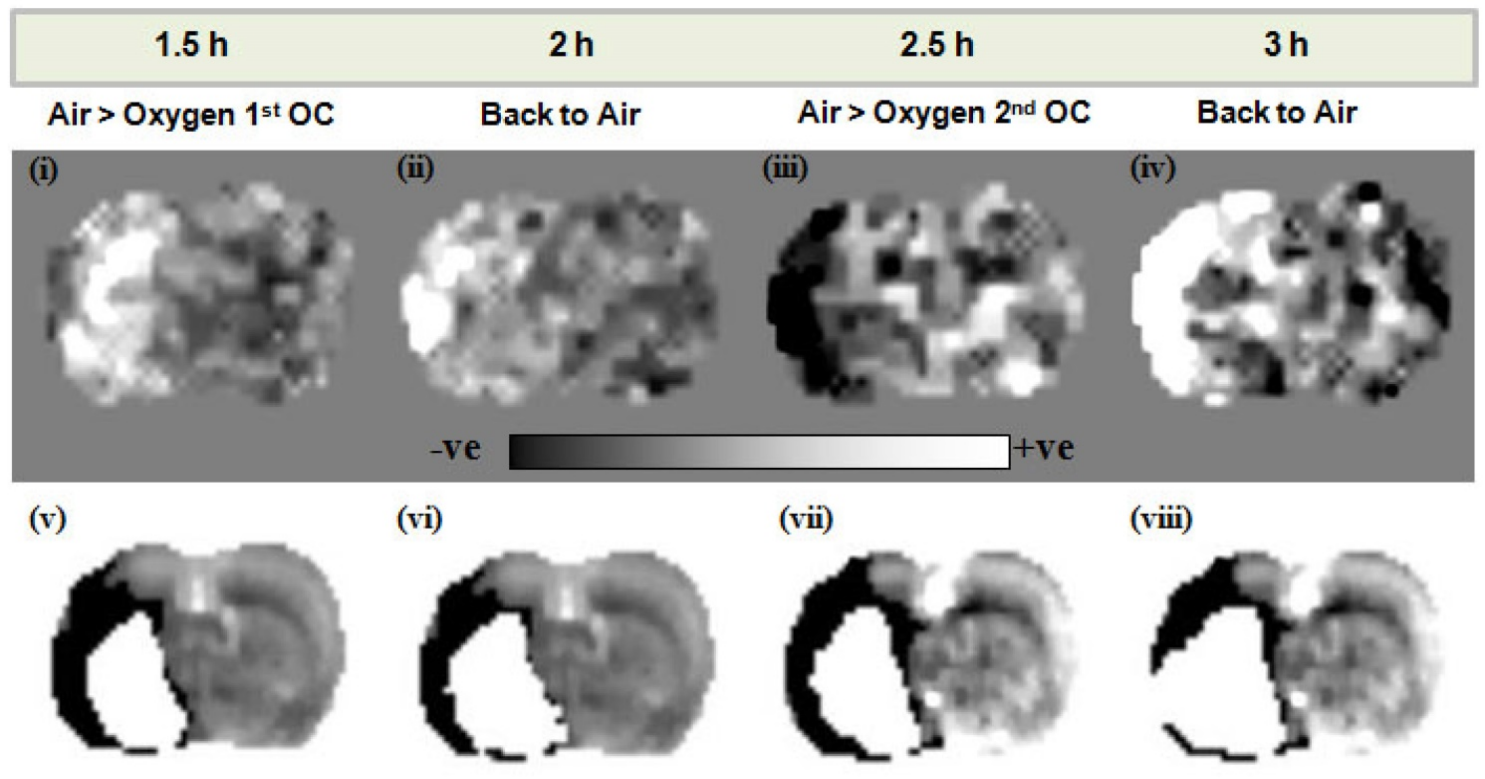
Lactate change aerobic (i, iii) and anaerobic (ii, iv) maps following administration of O-PFC (4.5 mL/kg) with time matched DWI/PI mismatch images (v-viii) for the first (1.5 h post-MCAO) and second (2.5 h post-MCAO) OCs. Regions of increased (white regions) or decreased (black regions) lactate evident in images i-iv were similar to but not exactly matching the region of DWI-PI mismatch shown in black in images v-viii. The white region in images v-viii represents the thresholded ADC lesion representing the ischaemic core. ADC: apparent diffusion coefficient; DWI: diffusion-weighted imaging; MCAO: middle cerebral artery occlusion; OC: oxygen challenge; PI: perfusion imaging.

Elevated lactate levels were detected in the ischaemic hemisphere following stroke in Study 2. The O-PFC and hyperoxic challenge resulted in a decrease in ischaemia-induced lactate on lactate change maps in all animals. In the representative scans in Figure 10, a decrease in tissue lactate can be observed in response to an OC at ~2.5 h following MCAO (Figure 10 iii). In this example the region of lactate change closely approximates but is different to the DWI/PI mismatch region in the temporally matched DWI/PI image (Figure 10 vii). On returning back to normoxic ventilation, the lactate change map indicates an increase in lactate within this region (Figure 10 iv) and the thresholded ADC derived lesion has increased in size (Figure 10 viii). These regions of change in tissue lactate indicate that this hypoperfused tissue is still metabolically active as it retains the ability to switch between aerobic and anaerobic metabolism.

##### Evidence of ongoing glucose use in regions identified as penumbra by GOLD imaging techniques

**Figure 11:**
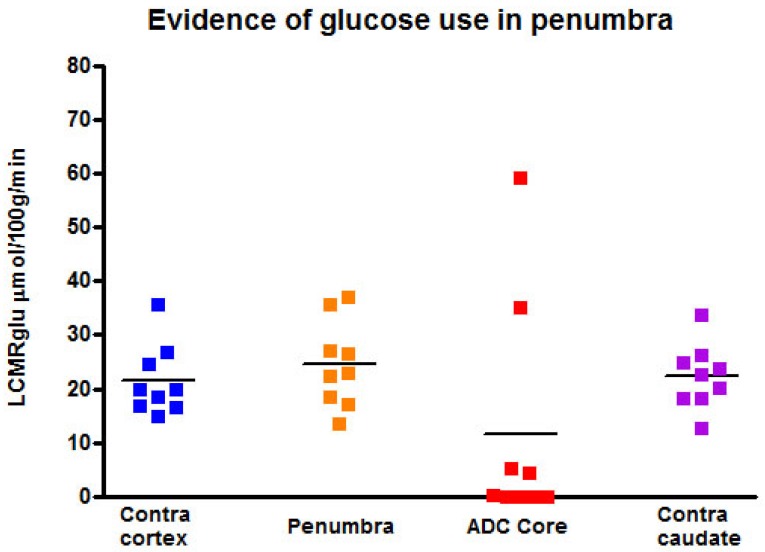
Local cerebral glucose utilisation (LCMRglu) in regions of interest generated following the completion of the later T2*OC and Lactate Change OC maps (~3 h following MCAO). Individual animal data shown with horizontal line representing mean. MCAO: middle cerebral artery occlusion; OC: oxygen challenge.

[^14^C]2-deoxyglucose autoradiography confirmed maintained glucose use within the T_2_* and lactate change OC-defined penumbra. The ROI on the [^14^C]2-DG autoradiogram which mirrored penumbra defined by T_2_* signal change and lactate change maps displayed a level of glucose metabolism that was not different from the contralateral ROI (24.5 ± 8.0 mmol per 100 g per minute compared with 21.5 ± 6.6 mmol per 100 g per minute in contralateral cortex; Figure 11). In contrast, glucose metabolism was markedly decreased in the ADC-defined ischaemic core in the majority of animals. In two animals where 100% O_2_ hyperoxia was maintained throughout the duration of the 2-DG autoradiography protocol, elevated glucose use was identified within the ADC core ROI (Figure 11), in an area distinct from the hyperglycolytic band previously described 15. This is an interesting observation, which could support the use of prolonged periods of hyperoxia following O-PFC administration as a potential therapeutic approach in acute ischaemic stroke.

## Discussion

There has been an increased effort over recent years to develop advanced acute stroke brain imaging techniques that can metabolically delineate the ischaemic penumbra from the irreversibly damaged core and benign oligaemia (hypoperfused tissue destined to survive). This would enable significant improvements in the management of acute stroke patients by differentiating patients with viable brain tissue (therapeutic target) irrespective of time from stroke onset, from those who have largely irreversible damage at presentation and who could therefore be exposed to additional risk from reperfusion therapy. In this paper we have presented further preclinical validation of two complementary diagnostic MR imaging techniques, which when combined with intravenous O-PFC and OC, (GOLD imaging), have the potential to identify metabolically active penumbra in rodent models of acute stroke. Further studies (unpublished) demonstrating a concomitant therapeutic benefit of O-PFC and hyperoxia supports the true theranostic potential of this novel technology, for which initial clinical trials in acute stroke patients are planned for late 2017.

The first studies to metabolically map the ischaemic penumbra following acute stroke used PET 28, the gold standard method identifying metabolically active brain tissue with reduced cerebral blood flow, an increased oxygen extraction fraction and a preserved cerebral metabolic rate of oxygen 29. However due to a number of factors including lack of availability, extended scanning time and radiation exposure, PET has not been adopted for clinical use in acute stroke. Focus has therefore shifted towards the use of other, more widely available imaging modalities (MRI, CT Perfusion (CTP)) in the pursuit of providing physicians with a diagnostic tool to identify patients with metabolically active penumbra 30. However, these techniques, which rely on selecting threshold values to define the DWI lesion and/or the perfusion deficit, do not provide direct information on metabolic activity. Neither have been validated and accepted for clinical use for routine for penumbra imaging, possibly because of a lack of consensus on threshold setting to accurately compartmentalise brain tissue into irreversibly damaged core, penumbra and benign oligaema. Indeed MR perfusion imaging, integral to DWI/PI mismatch, has been shown to be inherently poor in discriminating hypoperfused penumbra from benign oligaemia 10,11. Furthermore, uncertainty remains around whether the thresholded DWI lesion truly represents irreversibly damaged core tissue since early DWI lesions have the capacity to display penumbral characteristics with evidence of reversal in response to acute reperfusion 31,32. Early clinical studies (DIAS and DEDAS) investigating the thrombolytic drug desmoteplase, suggested that patients selected with DWI/PI mismatch demonstrated higher rates of reperfusion upon treatment initiated up to 9 h following symptom onset 33,34. The subsequent DIAS II trial was the first phase 3 trial to include an estimate of DWI/PI mismatch as an indicator of penumbra in the inclusion criteria 30. However the outcome of this trial was negative and much of the attention following this disappointing result, focussed on the criteria used to define penumbra. The DIAS II trial used a MR perfusion deficit that was ~20% larger compared against a time matched DWI lesion considered to be ischaemic core. Some patients were also selected in this study on the basis of a CTP deficit. In both instances this was done without applying appropriate perfusion thresholds to define penumbra. This combined with pooling of patients selected by both MR perfusion and CTP undoubtedly added to increased heterogeneity in the patients included and was retrospectively recognised to be inadequate in accurately selecting a patient population with true penumbra 35.

Likewise CTP demonstrates a high degree of heterogeneity in the acquisition protocols and post-processing software and this lack of standardisation has resulted in the lack of established validated thresholds for the quantitative definition of the ischaemic penumbra 10,11. Even with accurate quantitative measurement of blood flow, it is not possible to prospectively set a perfusion threshold for the ischaemic penumbra, as the amount of blood flow required to maintain tissue viability is not fixed but varies over time 36,37. Using the time to peak and mean transit times as surrogate measures of perfusion to identify the penumbra lack precision, as intracranial collateral blood flow pathways vary across individuals and brain regions. A recent systematic review incorporating more than 2000 patients across 27 studies was carried out to assess the diagnostic accuracy of CTP in acute ischemic stroke 38. It concluded that more high quality evidence is required for CTP to be used as a diagnostic tool that can reliably inform on selection of stroke patients for treatment. A lack of agreement on CTP processing parameters, biologically significant perfusion parameters and thresholds applied for decision making were highlighted. All of this adds to variability in the sensitivity of the technique and increases the uncertainty in the accuracy of its use in directing important treatment decisions 39,40,41.

MR perfusion and CTP were also used to select patients in clinical trials assessing endovascular therapy. In the MR RESCUE trial a lack of correlation between penumbral pattern on neuroimaging and outcome in terms of benefit from endovascular therapy was demonstrated 42. As discussed in a recent meta-analysis 43, further clinical studies on endovascular recanalisation therapy have proven the technique to be of benefit in patients with large vessel ischaemic stroke. In general, the trials included within this meta-analysis utilised brain imaging such as CTP to exclude patients with large ischaemic cores who would have been less likely to have a good outcome. Current trials on thrombolytic therapy (ECASS-4) and endovascular thrombectomy (DEFUSE 3) are further investigating the use of MR and CT to select patients on the basis of penumbral patterns from brain imaging [(44]; NCT02586415).

Therefore it is clear that, at present, these techniques require further refinement and optimisation to identify potential responders to reperfusion therapy beyond the currently recommended time windows. Furthermore, as they don't provide a metabolic approximation of penumbra their clinical utility in this regard will remain open to question.

The objective of the studies presented here was to advance the development of two complementary metabolically MRI based neuroimaging techniques (T2* and Lactate Change sequences combined with OC and O-PFC = GOLD imaging) for diagnostic use in the acute stroke setting to determine the presence of salvageable penumbra based on the metabolic status of this tissue.

Proof-of-concept for detection of penumbra was initially established for both techniques using 100% oxygen alone in rodent stroke models 13,14 with the T2*OC already translated to 3T clinical scanners and tested in healthy volunteers and stroke patients 18. However, signal-to-noise was poor with the T2*OC on translation to the clinical scanner and 100% oxygen inhalation resulted in spatial distortion of T2*-weighted contrast and significant artefacts within the human forebrain (due to paramagnetic oxygen in airways and nasal sinuses).

It is “change” in tissue lactate to an oxygen challenge, not absolute lactate levels, that are important in assessing “metabolic integrity” (or salvageability). Our group previously developed the novel method of lactate change MRI, which is based on imaging changes in brain tissue lactate levels in response to hyperoxic challenges 14. This metabolic method reflects capacity of tissue to switch between anaerobic to aerobic metabolism when additional oxygen is delivered. This would happen only if the mitochondria are functioning and so can produce the necessary ATP (38 ATPs generated with aerobic metabolism vs 2 ATPs with anaerobic metabolism for every molecule of glucose) to maintain cell function. Cell energetics are key to the survival of ischaemic tissues. Therefore, by identifying tissues with anaerobic metabolism but with a capacity to utilise oxygen and capability for aerobic metabolism, OC lactate change imaging, identifies salvageable penumbral tissue. Lactate Change OC proof of principle was also demonstrated with 100% oxygen alone but again there were limitations regarding sensitivity and the need for long scan times to observe lactate change 14.

The primary aim of Study 1 was to evaluate whether addition of a PFC improved the sensitivity and reduced the time for the Lactate Change OC, making the technique an applicable tool in the clinical setting. The data presented in Figures 3-5 provide evidence of further improvements in the technique through prior administration of b-PFC with changes in tissue lactate specific to the penumbra voxel being seen following hyperoxic challenge in the animals receiving b-PFC. During hyperoxia, lactate levels in the penumbra voxel showed a trend for an overall increase in the control saline group (3 animals showed increase, 2 no change and 3 showed a decrease). This is indicative of a continued switch to anaerobic metabolism in the hypoperfused penumbra tissue, resulting in further increases in tissue lactate levels. It also confirms that normobaric hyperoxia alone is not always sufficient in this model to improve oxygenation of the penumbra to the extent that aerobic metabolism is restored. However, in the group of animals receiving b-PFC, lactate levels in the penumbra voxel were decreased in all animals and significantly reduced after 10 minutes of hyperoxia. It is known that PFC micelles are 1/30^th^ - 1/40^th^ the size of red blood cells (diameter of about 200 nanometers) and are therefore able to penetrate the smallest blood vessels, enabling oxygen delivery to ischaemic tissues. This along with an improved extraction ratio of oxygen from PFC emulsions when compared to haemoglobin 45 could improve oxygen delivery such that there is a switch back to aerobic metabolism with the resulting decrease in tissue lactate. This provides validation that intravenous PFC increases the sensitivity of our Lactate Change OC diagnostic technique in detection of penumbra compared to normobaric hyperoxia alone and has the potential to translate into clinical application, as the change can be imaged with shorter scanning time. No changes in lactate levels were observed in either the ischaemic core or contralateral voxels in either group. This is likely to be due to tissue within the ischaemic core being unable to recover aerobic metabolism despite the increased delivery of oxygen during hyperoxia (i.e. tissue is no longer metabolically active). The lack of effect in the contralateral caudate nucleus is likely to be a reflection of little or no increase in lactate level within this region of the brain acutely following stroke.

We next investigated the potential of combining the metabolic GOLD imaging techniques (T2*OC and Lactate Change OC) within a single scanning protocol using O-PFC. The protocol was designed such that T2*OC and Lactate Change OC imaging could be applied concurrently in the same scanning session in a way that would most likely mimic the intended clinical utility of these imaging techniques in the acute stroke setting.

For lactate change imaging, the example shown in Figure 10 indicates that during the initial OC, at ~1.5 h post-MCAO there is a small increase rather than decrease in lactate within the ipsilateral hemisphere (Figure 10 i) in a region close to the ischaemic core when comparing to the time-matched ADC lesion (Figure 10 v). It may be that in this particular animal tissue lactate levels are rapidly increasing at this time, in response to the ischaemic insult, making it difficult to image a decrease in response to hyperoxia. On returning to normoxia a further increase in tissue lactate levels was observed in the ipsilateral hemisphere (Figure 10 ii). However, in response to the second OC at ~2.5 h post-MCAO there was a clear decrease in tissue lactate observed in an ipsilateral region, similar to but not identical in size and location to DWI/PI mismatch (Figure 10 iii and vii). This decrease in tissue lactate provides evidence of tissues that are hypoperfused and under metabolic stress but that retain the capacity to respond to increased oxygen delivery by switching to aerobic metabolism. The earliest sign of cellular injury is neuronal swelling or shrinkage and micro-vacuolation of the cytoplasm, which is associated with mitochondrial swelling. These changes are reversible. Once the mitochondrial membranes rupture then the process is irreversible. The membrane potentials are maintained by ion pumps, the most important being the Na^+^/K^+^ ATPase and these require energy. Anaerobic metabolism of glucose only provides 2 ATPs from a molecule of glucose but with aerobic metabolism one molecule of glucose can provide additional 36 ATPs. The energy produced by anaerobic metabolism cannot sustain the membrane potentials and this lack of energy ultimately results in cell death. As long as the mitochondria are functional and able to utilise oxygen to generate ATP, the neuron will have the energy to survive. Therefore the ischaemic penumbra represents hypo-perfused tissue displaying anaerobic metabolism due to lack of oxygen, which still has the potential for aerobic metabolism and so is potentially salvageable.

The results presented in Study 2 utilising spectroscopic imaging of lactate to map lactate change in response to hyperoxic challenge following O-PFC administration, corroborate with the results from Study 1 where b-PFC was shown to improve the possibility to detect decreases in the lactate spectral peak. Together these findings substantiate an enhancement by PFC to delineate metabolically active penumbra using this technique. Furthermore the ability to detect regions of lactate change in all animals analysed in Study 2 signifies an improvement in the sensitivity to detect lactate change, through the addition of O-PFC emulsion. Previously published findings reported a decrease in tissue lactate in only a subset of animals when using 100% O_2_ alone 14.

To address limitations encountered during preliminary translation of the T2*OC technique an intravenous PFC was administered prior to OC with lower oxygen levels. Following administration of our b-PFC, lower level hyperoxia (40-50% O_2_) significantly enhanced T2* signal change in the ischaemic penumbra ROI compared with lower oxygen levels alone 21. Study 2 results provide confirmation of this finding with intravenous O-PFC emulsion, which is the PFC product intended for subsequent clinical studies. The magnitude of T2* signal change in penumbra was consistent with our previous study. This validates the use of O-PFC in combination with low-level hyperoxia for enhancing the T2*OC method, which should overcome both artefact and signal to noise issues encountered in early clinical studies. Indeed upon quantification of the region identified as penumbra by thresholded T_2_*OC signal change maps, there was no difference in the volume detected by either 50% or 100% oxygen providing further support for increased sensitivity at lower levels of inhaled oxygen with the addition of O-PFC. It was however notable from Study 2 that the volume of the region detected as penumbra during the later T_2_*OC at ~2.5 h post-MCAO, was significantly smaller than that detected at the earlier time point. This indicates a progression of the ischaemic damage and incorporation of penumbral tissue into ischaemic core between the two OCs when animals were ventilated with air. This provides additional validation that the technique identifies ischaemic penumbra and also that in the absence of hyperoxia, O-PFC was limited in its capacity to maintain penumbra viability.

**Figure 12:**
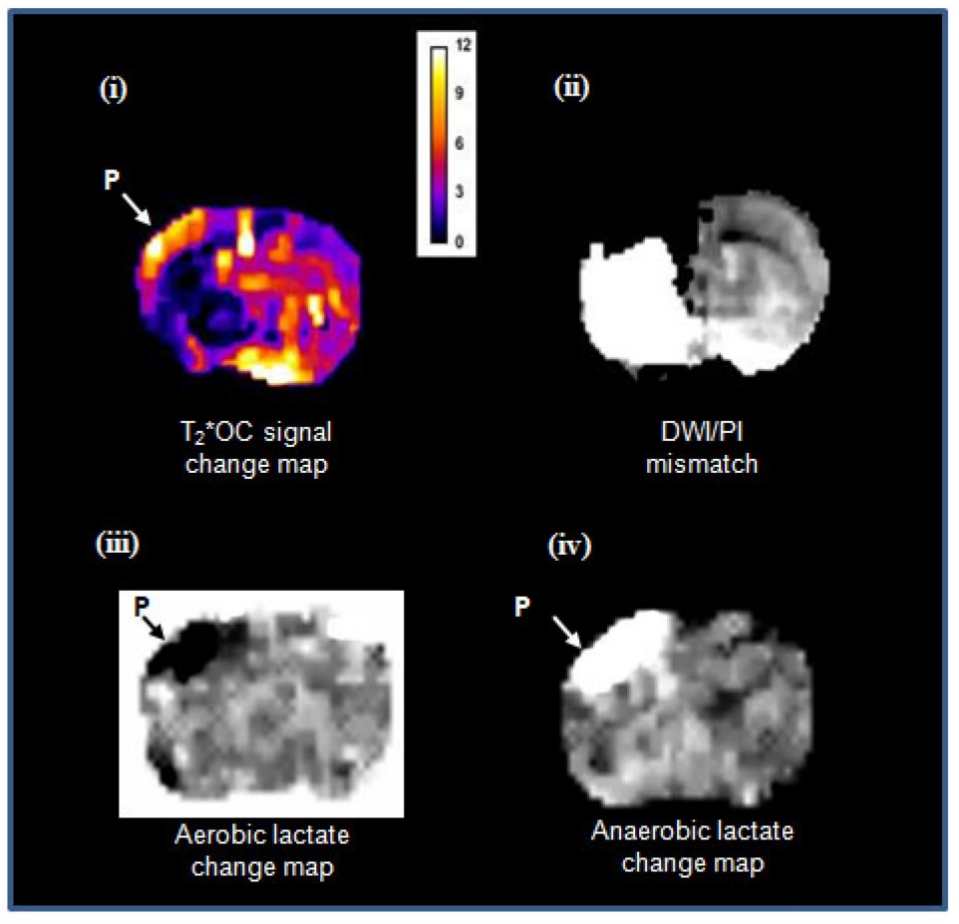
Matched images illustrating both T_2_*OC and Lactate Change OC GOLD penumbra techniques working simultaneously in a representative animal following administration of O-PFC (~4.5 mL/kg). (i) T_2_* signal change map to 50% OC at ~2.5 h post MCAO. (ii) corresponding time-matched DWI/PI mismatch. (iii) Aerobic lactate change map in response to 50% OC at ~3 h post MCAO and (iv) anaerobic lactate change map in response to returning animal to normoxic ventilation at ~3 h post-MCAO. P, penumbra. DWI: diffusion-weighted imaging; GOLD: Glasgow Oxygen Level Dependent; MCAO: middle cerebral artery occlusion; OC: oxygen challenge; PI: perfusion imaging.

In meeting the objective of Study 2, both GOLD imaging techniques, T_2_*OC and Lactate Change OC, were successfully run within the same scanning protocol, working concurrently to identify penumbra following administration of O-PFC. Example images showing penumbra identified by both techniques running concurrently in the same animal are illustrated in Figure 12. It is evident that regions identified as penumbra on T_2_*OC (Figure 12 i) and Lactate Change OC maps (Figure 12 iii and iv) were not identical to each other or to the region defined by DWI/PI mismatch (Figure 12 ii). The T_2_*OC identifies hypoperfused tissues with oxygen utilisation and an increased oxygen extraction fraction whilst the Lactate Change OC imaging identified hypoperfused tissues displaying anaerobic metabolism but having the potential for aerobic metabolism. Both these techniques are assessing different aspects of metabolism and therefore it is not surprising they were not identical as shown in our study.

A final objective of this study was to provide validation of the combined techniques by confirming on-going tissue glucose metabolism in hypoperfused regions of the brain identified as penumbra by both T2* and lactate change OC. Terminal [^14^C] 2-deoxyglucose autoradiograms, confirmed glucose metabolism within both the T_2_*OC-defined and the Lactate Change OC identified penumbra. Therefore, both techniques are complementary and further studies will help define their individual advantages and disadvantages.

It was notable that when ventilation was switched back from 100% oxygen to air in Study 1 that lactate levels significantly increased in the saline group at 30 minutes while the decreased lactate levels were maintained below pre-hyperoxia baseline values in the b-PFC group. This suggests that animals given b-PFC could maintain aerobic metabolism in the penumbra voxel, presumably through more efficient delivery of oxygen to this tissue unlike the control animals receiving saline. Due to the small size of the PFC nanoparticles (35-45 times smaller than red blood cells) they have the capacity to reach the microcirculation beyond an occluding clot in any remaining plasma flow, or via collaterals, thereby improving delivery of oxygen to the compromised ischaemic penumbra. It has been shown that ischemia induces sustained contraction of pericytes on microvessels and thereby causes capillary constriction and obstruction of erythrocyte flow 46. PFC nanoparticles (~0.2 μm compared to ~7 μm for erythrocytes) could therefore improve delivery of oxygen within this compromised microcirculation.

Initial evidence for the ability of PFC to support the ischaemic penumbra and offer therapeutic potential following acute ischaemic stroke comes from analysis of lesion growth in Study 1. In addition to measuring lactate, a secondary aim of this study was to quantify the area of ischaemic damage from thresholded ADC maps during the period of hyperoxia and return to normoxia. It was shown that lesion expansion during this period was significantly smaller in the group of animals receiving b-PFC when compared to the group receiving saline prior to hyperoxia. This suggests that animals given b-PFC in combination with hyperoxia could maintain aerobic metabolism because of functioning mitochondria and the resulting cell energetics would support the survival of penumbral tissue.

In summary enhancement of both GOLD imaging techniques following intravenous O-PFC and hyperoxia has been demonstrated for the first time *in vivo*, working concurrently to identify the metabolic penumbra in a rodent stroke model. Regions identified as penumbra with both T_2_^*^OC and Lactate Change OC techniques displaying maintained glucose metabolism. Further development of co-registration and analysis software to provide a penumbra map on clinical MRI scanners is ongoing.

Successful translation of GOLD imaging would enable treatment decisions and recruitment into future acute stroke trials investigating efficacy endpoints to be based on metabolic status of the brain tissue independent of time from stroke onset. This could improve the safe use and extend the availability of current therapeutic options to many more acute stroke patients. We have also uncovered preliminary evidence for a potential therapeutic benefit of O-PFC plus hyperoxia during scanning, which could offer significant advantages over current penumbral imaging techniques by slowing the progression of ongoing ischaemic damage. Further therapeutic benefit could be realised by extending hyperoxia beyond the scanning period and this is the focus of additional research by our group. Therefore, through unique simultaneous diagnostic and therapeutic application in acute ischaemic stroke, GOLD imaging technology incorporating intravenous O-PFC and hyperoxia offers new hope in improving the management of acute stroke patients.

## Supplementary Material

Supplementary figures and tables.

## Abbreviations

ADC: apparent diffusion coefficient
AIS: acute ischaemic stroke
ASL: arterial spin labelling
AUC: area under the curve
BOLD: blood oxygen level dependent
b-PFC: bespoke perflurocarbon emulsion
CBF: cerebral blood flow
CT: computed tomography
DWI: diffusion-weighted imaging
CTP: CT perfusion
EPI: echo planar imaging
FiO_2_: fraction of inspired oxygen
GOLD: Glasgow Oxygen Level Dependent
LCMRglu: local cerebral glucose utilisation
MABP: mean arterial blood pressure
MCAO: middle cerebral artery occlusion
MRI: magnetic resonance imaging
MRS: magnetic resonance spectroscopy
OC: oxygen challenge
O-PFC: Oxycyte perfluorocarbon emulsion
PFC: perfluorocarbon
PI: perfusion imaging
PET: positron emission tomography
PRESS: point resolved spectroscopy sequence
rtPA: recombinant tissue plasminogen activator
ROI: region of interest
SD: standard deviation.
