# Supplementary material for: Perfluorocarbon Enhanced Glasgow Oxygen Level Dependent (GOLD) Magnetic Resonance Metabolic Imaging Identifies the Penumbra Following Acute Ischemic Stroke
Source: Theranostics. 2018 Feb 12;8(6):1706–22. doi: 10.7150/thno.21685 (PMC5858177; doi:10.7150/thno.21685)
Supplement: Supplementary file 1 — Supplementary figures and tables. [file thnov08p1706s1.pdf]

Supplementary Information

Perfluorocarbon Enhanced Glasgow Oxygen Level Dependent (GOLD) Magnetic Resonance Metabolic Imaging Identifies the Penumbra Following Acute Ischemic Stroke.

Graeme A Deuchar[1,3], David Brennan[2,3], William M Holmes[1], Martin Shaw[4], I Mhairi Macrae[1], Celestine Santosh,[2,3].

[1]Glasgow Experimental MRI Centre, Institute of Neuroscience and Psychology, College of Medical, Veterinary and Life Sciences, University of Glasgow; [2]Department of Neuroradiology, Institute of Neurological Sciences, Southern General Hospital, Glasgow, UK; [3]Aurum Biosciences Ltd, 20-23 Woodside Place, Glasgow, G3 7QL. [4]Clinical Physics, University of Glasgow, Western Infirmary, Glasgow, United Kingdom.

Address for correspondence:

Dr Graeme Deuchar,

Aurum Biosciences Ltd,

20-23 Woodside Place,

Glasgow, G3 7QL,

Scotland, UK

Tel: +44 141 582 1212

Email: gdeuchar@aurumbiosciences.com

	Saline (4.5 mL/kg)		Oxygen Challenge		b-PFC (4.5 mL/kg)		Oxygen Challenge	
	Baseline	During	Baseline	100% O_2	Baseline	During	Baseline	100% O_2
MABP (mmHg)	89.4 ± 4.4	91.0 ± 4.5*	86.6 ± 6.0	101.0 ± 5.4**	82.8 ± 5.6	89.7 ± 7.0**	78.6 ± 7.8	95.1 ±10.7**
PaO_2 (mmHg)			87.4 ± 7.7	322.6 ± 33.0**			89.5 ± 8.4	333.8 ± 36.4**
$PaCO_2$ (mmHg)			33.0 ± 6.1	38.5 ± 9.7			32.1 ± 7.0	36.0 ± 11.4

Table S1: Physiological parameters (n=6-9) before (baseline) and during either saline or b-PFC administration (4.5 mL/kg. i.v) and before and during hyperoxic oxygen challenge (100% O_2). Data are expressed as mean ± s.d. *p<0.05; **p<0.01, Significant increase in MABP and PaO2 compared to corresponding baseline value, Student's paired *t*-test.

	O-PFC (4.5 mL/kg)		1st Oxygen Challenge			2nd Oxygen Challenge		
	Baseline	During	Baseline	50% O_2	100% O_2	Baseline	50% O_2	100% O_2
MABP (mmHg)	92.1 ± 6.4	100.1 ± 8.9**	88.7 ± 6.6	101.8 ± 6.8**	102.6 ± 6.4**	93.0 ± 8.9	104.8 ± 8.2**	102.2 ± 9.8**
PaO_2 (mmHg)			87.8 ± 6.0		366.8 ± 35.1**	89.5 ± 8.4		333.8 ± 36.4**
$PaCO_2$ (mmHg)			41.0 ± 12.2		42.0 ± 8.3	41.0 ± 12.2		42.0 ± 8.3

Table S2: Physiological parameters (n=6-9) before (baseline) and during O-PFC administration (4.5 mL/kg. i.v) and before and during hyperoxic oxygen challenges (50% & 100% O_2). Data are expressed as mean ± s.d. **p<0.01. Significant increase in MABP and PaO2 compared to corresponding baseline value, Student's paired *t*-test.

Figure S1.

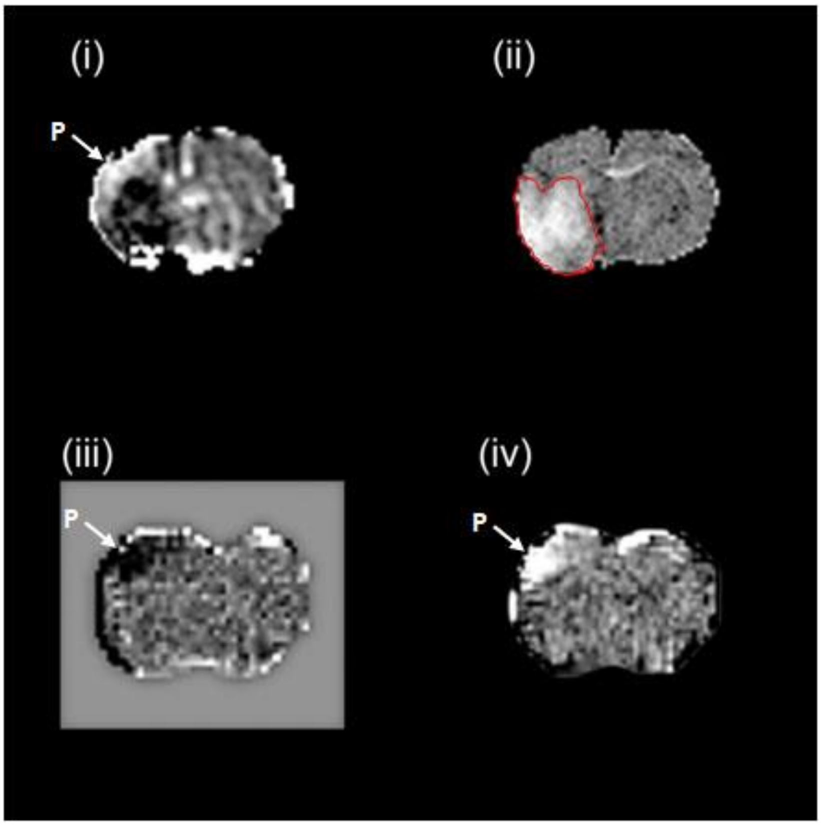

Figure S1. Four matching images demonstrating the automated image processing showing co-registered variants of the analyses performed in Study 2: (i) T2* signal change map (50% O_2), (ii) Autoradiography image with area of decreased glucose use outlined in red, (iii) Aerobic lactate change map (ventilation changed from air to O_2) and (iv) Anaerobic lactate change map (ventilation changed from O_2 back to air). P indicates penumbra.
